# Nutrient Accumulation Pattern in Mixtures of Wheat and Faba Bean Is Strongly Influenced by Cultivar Choice and Co-Existing Weeds

**DOI:** 10.3390/biology11050630

**Published:** 2022-04-20

**Authors:** James Ajal, Martin Weih

**Affiliations:** Department of Crop Production Ecology, Swedish University of Agricultural Sciences, P.O. Box 7043, SE-75007 Uppsala, Sweden; martin.weih@slu.se

**Keywords:** scaling exponent, cereal–legume, weed competition, species mixtures, nutrient accumulation, plant–plant interaction

## Abstract

**Simple Summary:**

Growing a mixture of two or more crop species, particularly cereals and legumes, can enhance resource use efficiency for growth-limiting resources, such as nutrients. We evaluated the patterns of nutrient accumulation efficiency in different cultivars of faba bean and wheat grown in mixtures with and without the presence of weeds in a growth container experiment. The cultivar used in the mixture determined the amount of nitrogen accumulated by the legumes, but cereals generally accumulated more nitrogen in the mixtures than when grown as sole crops. Competition from weeds resulted in lower nitrogen accumulation in the crop plants, and plant neighbor identity affected the accumulation of other nutrients relative to the accumulation of nitrogen and phosphorus in the plants. Cultivar choice is therefore important for resource limitation and thereby the growth performance of plants grown in mixtures.

**Abstract:**

Cereal–legume mixtures are often associated with higher yields than the components grown as sole crops, but the underlying mechanisms are unclear. The study aims to evaluate how different cultivars in a two-species wheat–faba bean mixture influence above- and below-ground nitrogen (N) accumulation in the plant biomass, whether crop mixing affected the accumulation of other nutrients relative to the accumulation of N and phosphorus (P), and how the nutrient accumulation pattern in sole crops and mixtures is influenced by weed competition. Using a growth container experiment, we investigate nutrient accumulation patterns on specific wheat and faba bean cultivars grown as sole crops and mixtures, and with and without weed competition. We found that cereals in the mixture accumulated more N than in the sole crops, and the cultivar used influenced biomass accumulation in the legumes. Competition from weeds reduced the amount of plant N pools accumulated in the crop plant biomass. Based on stoichiometric scaling exponents, the plant neighbor affected the accumulation of other nutrients relative to the accumulation of N and P. These results are relevant for species and cultivar selection, all of which are important prerequisites for maximizing mixture performance.

## 1. Introduction

Increasing the diversity of crop systems could enhance and stabilize crop yields while increasing ecological sustainability. Legume-supported mixtures are often exploited to achieve increased nutrient uptake, and hence reduce the requirement for external fertilizer input [1,2]. While the realized benefits of growing mixtures have previously been associated with low input systems, recent studies have shown that intercropping could realize similar benefits also in high-input systems [2,3]. Species mixtures, especially those of cereal–legumes, make use of positive plant–plant interactions, which facilitate a complementary use of resources, such as light and nutrients [4,5]. In addition, mixtures can deliver services that may not be realized in sole crops. For example, legumes are weak competitors against weeds, but growing them as mixtures with cereals significantly improves the weed suppressive ability of the crop [6,7].

Based on ecological theory, it should be possible to realize the benefits of cereals and legumes grown as mixtures irrespective of the specific crop component used. In practice, specific cereal–legume combinations are commonly preferred, which could be related to cropping regimes that either result in the buildup of diseases [8], or beneficial fungal and bacterial communities in the soil [9]. Mixtures of wheat (*Triticum aestivum* L.) and faba bean (*Vicia faba* L.) are among the most commonly grown cereal–legume mixtures in Europe and offer great potential for use in fodder production. The selection of cultivars to include in these mixtures is crucial in designing productive mixtures, as specific cultivar traits may influence plant nitrogen (N) dynamics and thus the productivity of the mixture [10]. This is particularly important if the cultivars in the mixture differ in traits that influence competition between the species and hence increase spatial complementarity, for example, the shoot architecture, which may affect canopy structure and light interception [11], or the root length density, which is important for below-ground interactions and the uptake of nutrients that limit plant growth [12].

Nitrogen and phosphorus (P) are considered the most important for limiting plant growth [13,14]. For this reason, N and P are the center of attention for most studies that focus on nutrient use [15], and specifically in cereal–legume mixtures [1,16,17,18]. However, other nutrients play a crucial role in different plant processes and can co-limit plant growth. For example, [15] found evidence for co-limitation by magnesium (Mg) and P in field-grown wheat using scaling exponents. Scaling exponents determine the balance between different nutrients and how this balance is affected by different environmental factors. In this study, we use scaling exponents to quantify the stoichiometric relationship of N and P with respect to a set of other macronutrients, calcium (Ca), potassium (K), Mg, and sulphur (S). A scaling exponent >1 indicates that the relative accumulation of the other nutrients in the plant biomass occurs at a greater rate compared to the combined accumulation of N and P, and a scaling exponent <1 indicates that the relative accumulation of the other nutrients occurs at a lower rate compared to the accumulation of N and P [13]. In that context, resource complementarity is an important mechanism positively influencing plant interaction in mixtures [5]. Although complementarity in mixtures is mostly known to occur for N and P, we expect that the same may apply also for other (co-) limiting nutrients. Moreover, due to plant–plant interactions, the presence of some plants can alter the nutrient concentrations of other plants growing in the vicinity, for example, as shown for P availability in maize grown with mixed species of weeds [19].

Using cultivars of wheat and faba bean grown in sole crops and mixtures as our model system, we aim to evaluate how the different cultivars in the two-species wheat–faba-bean mixtures influence above- and below-ground N accumulation in the plant biomass, and whether crop mixing results in different scaling exponents (N and P vs. other macronutrients) than in the sole crops. In addition, we aim to investigate how the N accumulation and scaling exponents in sole crops and mixtures are influenced by weed competition. The aims are evaluated by means of a semi-controlled growth container experiment in Uppsala during the 2019 growing season. Specifically, we hypothesized that: *(H1)* the wheat grown in a mixture with faba bean accumulates more N and biomass in the shoots and roots than when grown in the sole crop, as a result of increased N fixation from the legumes and the high competitiveness of the cereals; *(H2)* above- and below-ground N accumulation depends on the specific composition (i.e., cultivar choice) of the mixture; *(H3)* resource competition between the crop and weeds results in lower accumulated plant N pool and grain yield of the crops; and *(H4)* scaling exponents differ between sole crops and the same crop grown in mixture, and are affected by the presence of weeds.

## 2. Materials and Methods

### 2.1. Plant Material

Two cultivars (cv.) of spring wheat (*Triticum aestivum* L.), cv. ‘Diskett’ and ‘KWS Alderon’, and faba bean (*Vicia faba* L.), cv. ‘Boxer’ and ‘Fuego’, were grown as sole crops and in wheat–faba-bean mixtures. The wheat cv. ‘KWS Alderon’ is hereafter referred to as ‘Alderon’. All seed material was provided by Lantmännen Lantbruk, Svalöv, Sweden. The cultivars and species combinations were selected as they were previously grown under field conditions in Sweden and have contrasting characteristics. Specifically, the cereals ‘Alderon’ and ‘Diskett’ showed contrasting N accumulation, with ‘Alderon’ having higher N uptake and N accumulation efficiencies than ‘Diskett’ when grown in mixtures with faba bean in one of the years [10]. In addition to the cultivars used in the different species combinations, the various sole crops and mixtures were grown with and without a weed species (*Chenopodium album* L.), which commonly occurs in agricultural fields in the region, and has been characterized as highly responsive to the addition of nutrient fertilization [7].

### 2.2. Experimental Setup

**Figure 1:**
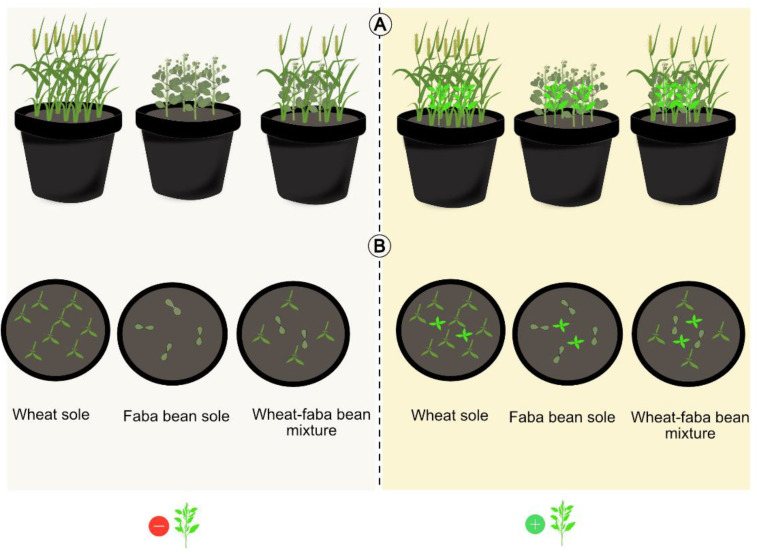
Illustration of a pot experimental treatment including wheat and faba bean with each crop grown as sole crop and in mixture. In half of the pots, the crop plants were grown with the weed *C. album* (+weed) and the other half without the weed treatment (−weed). Two cultivars of the cereal (cv. ‘Diskett’ and ‘Alderon’) and legume (cv. ‘Boxer’ and ‘Fuego’) were used in each case. (**A**) is a two-dimensional representation of the treatments and (**B**) represents the aerial view of the pots, showing the planting design during the early growth stages.

The experiment was conducted in Ultuna, Uppsala, Central Sweden (59°49′01.3″ N 17°39′26.0″ E) under semi-controlled conditions with plants exposed to similar weather conditions to those experienced outdoor or in the field, between 13 May–28 August 2019 (Figure S1). The experiment was arranged in a split-plot design with weed treatment as the main plots and the species combinations in sole crops and mixtures as subplots. Each block of 17 pots comprised (a) 2 cultivars of faba bean and wheat in sole crops and 2-species mixtures of the different cultivars grown together with weeds (+weed) and (b) 2 cultivars of faba bean and wheat in sole crops and 2-species mixtures of the different cultivars grown without weeds (−weed). An additional pot containing only weeds was included in each block. The experiment was replicated 8 times resulting in a total number of 136 pots. The growth medium was composed of mineral soil of 45% sand, 25% gravel, 18% coarse silt, 5% fine silt, and 5% clay. The pH of the mineral soil was 7.6, cation exchange capacity (CEC) was approx. 5.3 meq/100 g, and base saturation was >80%. The mineral soil was placed in plastic pots measuring 26 cm in diameter, 21.1 cm in height, and 7.5 L in volume. Mineral soil was chosen as it facilitated root washing for below-ground assessments. Seeds of wheat and faba bean were placed in Petri dishes with dump filter paper and left at room temperature for 48 h. Only pre-germinated seeds were used to ensure 100% seedling survival at the initial growth stage. The pre-germinated seedlings of each plant species and cultivar were transplanted to the pots. Based on the different growth characteristics of the two plant species, which determine space occupancy in a pot, eight and four seedlings were transplanted for wheat and faba bean, respectively, in the sole-crop pots, while the mixtures contained half the plant population of each species grown as a sole crop (Figure 1). Seven days after the planting (DAP) of the crop plants, *C. album* seedlings were transplanted to the respective pots with weed treatment (+weed). The *C. album* seedlings used in the experiment were of uniform size and selected from a weed population growing naturally in the vicinity of the experiment. The weeds were added in an additive design to pots that were identical to the ones without weed treatment (−weed). The plants were supplied with a uniform concentration of a 2 mL L^−1^ nutrient solution with a pH of 3.0. At the seedling stage, 100 mL of the nutrient solution (of concentration 2 mL L^−1^) was applied per pot once a week for the first four weeks. With increased crop demand during the growth period, 200 mL per pot was applied twice a week, and after crop flowering, 200 m of the nutrient solution was applied once a week until harvest. The nutrient solution was prepared from a balanced, complete fertilizer, ‘Wallco’ (Cederroth International, Upplands Väsby, Sweden), with NH_4_^+^ and NO_3_^−^ N in the proportion 2:3 and containing (g L^−1^): 51 N, 10 P, 43 K, 4 S, 3 Ca, 4 Mg, 0.17 Fe, 0.20 Mn, 0.10 B, 0.03 Zn, 0.015 Cu and 0.004 Mo. Watering was conducted when necessary to maintain a sufficient water supply for plant growth.

### 2.3. Measurements and Sampling

Phenological measurements were taken throughout the growing period, and periodic destructive samplings were conducted at crop flowering and maturity of the cereals. Whole-plant harvests were conducted at 78 DAP (wheat flowering) and at 107 DAP (wheat maturity) to determine above- and below-ground biomass accumulation. Half of the total number of blocks (4 blocks) were randomly assigned to destructive harvests at each vegetative sampling point. All biomass samples were dried at 70 °C for 48 h. For the plants harvested at crop flowering, the relative concentrations of N and the macronutrients Ca, K, Mg, P, and S were analyzed in the biomass of the different species. We focused on the dynamics of both N and macronutrients at the flowering stage because flowering marks an important transition from vegetative to reproductive phases in flowering plants. At maturity, only N was analyzed, but separately for the vegetative plant parts and grains. The nutrient contents in the biomass were used to calculate the nutrient concentrations and pools in the different plant parts grown in the sole crops and different mixture combinations, with and without weeds. We were unable to separate the roots of the different species in the mixtures, and the biomass and N accumulation in the roots of each component in the mixtures were therefore calculated based on the proportions of shoot biomass and N accumulation observed in the corresponding sole crops. This procedure is based on the assumption that the proportion of above- and below-ground accumulation of each species is similar in the sole crops and the same crop grown in a mixture.

### 2.4. Calculation of Nitrogen Accumulation Efficiency

Nitrogen accumulation efficiency (NAE) and its components were used to evaluate N acquisition and conversion of the cereals and legumes grown as sole crops and in mixtures, with and without the presence of weeds. The evaluations were based on the formula: NAE = U_N_ × E_N,g_ × C_N,g_ [20,21]. U_N_, the mean N uptake efficiency, is the component that represents the mean N uptake of the plant during the growth period and accounts for the N acquisition aspect of the plant per unit N in the sown seed. E_N,g_ is the grain specific N efficiency, calculated as a ratio of grain biomass at harvest and mean plant N content during the entire growth period. The grain specific N efficiency represents the N conversion aspect of the plant. C_N,g_ is the grain N concentration: the ratio of grain N content and grain biomass produced at harvest.

### 2.5. Calculations of Scaling Exponents for Nutrient Concentrations

A niche volume concept was applied to analyze the relationships between the vegetative tissue concentrations of N and P (expressed as their volume, VNP) and the concentrations of other macronutrients (VOth; Ca, K, Mg, S) [13]. Thus, based on the scaling relationship VOth = β(VNP)^α^, a faster increase in VOth in relation to VNP is reflected by scaling exponents (α) > 1, whilst a slower increase in VOth in proportion to VNP is reflected by scaling exponents < 1. Calculations of scaling exponents were based on data from the flowering stage only. All regressions for the stoichiometric volumes were calculated as reduced major axes (RMA; SPSS version 26) using ln-transformed values as is conventional in this type of allometric analyses [22].

### 2.6. Statistical Analysis

Apart from the calculations of scaling exponents, all other statistical analyses were performed in R version 4.0.2 (R Core Team, 2020). Before analysis, data were checked to ensure that the assumptions for normal distribution and equal variances were met. Data that did not meet the assumption for normality were log-transformed (log_e_) before analysis. Linear mixed-effects models (nlme Package: ‘*lme*’ by Pinheiro et al. [23]) were used, with each species analyzed separately for each sampling occasion. Mixture treatment (Mix.Sole), the cultivar in the mixture (Cultivar) and the weed treatment (Weed) were treated as fixed effects, while Weed nested within block as random effects. The calculation for NAE was based on data from both flowering and maturity, and instead of evaluating the different species depending on the mixture and weed treatments within each block, species were grouped based on the treatment for the whole experiment. Therefore, the model structure was similar as above (i.e., Cultivar, Mix.Sole and Weed as fixed effects), but the assigned treatment (Treatment) was regarded as random effect.

## 3. Results

### 3.1. Differences between Sole and Mixed Culture Were Mostly Related to N Uptake Efficiency, Not N Conversion Efficiency

**Figure 2:**
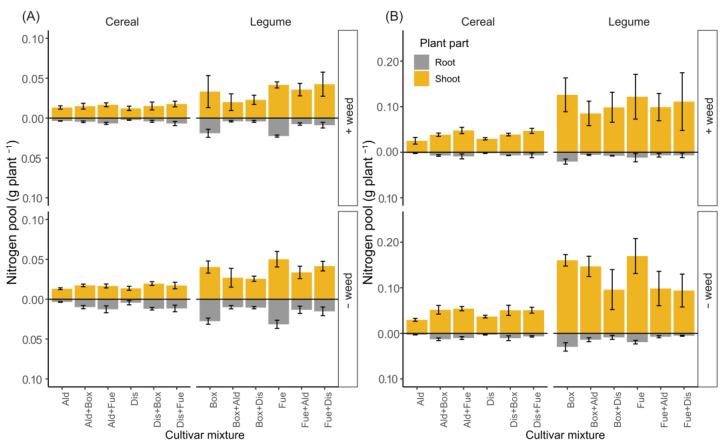
Above and below ground mean plant N pools for different wheat and faba bean cultivars pot-grown as sole crops and in mixtures, and sampled at flowering (**A**) and maturity (**B**). Each cultivar (Ald, ‘Alderon’; Dis, ‘Diskett’; Box, ‘Boxer’; and Fue, ‘Fuego’) was grown as a sole crop and in mixture, with (+) and without (−) the weed *C. album*. Above-ground N pools were calculated from leaves, stems and heads/pods (grains at maturity). Note the different scales in (**A**,**B**). The error bars represent ± 1SD.

**Figure 3:**
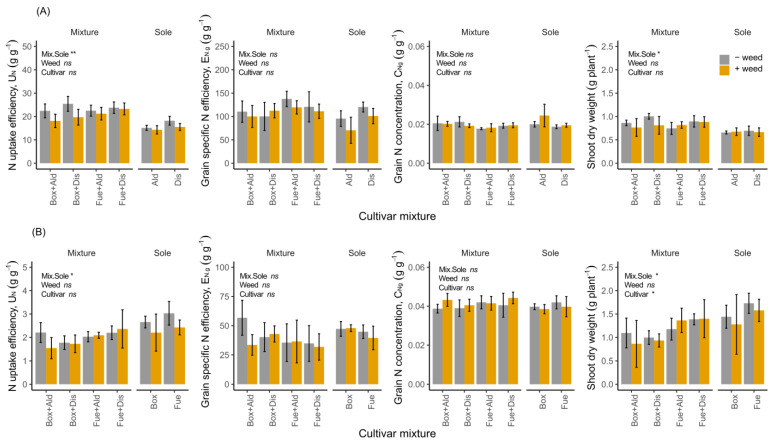
Nitrogen accumulation efficiency (NAE) components for cereals (**A**) and legumes (**B**), showing N uptake efficiency (U_N_), grain specific N efficiency, E_N,g_), grain N concentration (C_N,g_) and shoot dry weight at flowering. The different cereal and legume cultivars were pot-grown as sole crops and mixtures, with (+weed) and without (−weed) *C. album*. The full cultivar names are the same as in Figure 2. Symbols show ANOVA results with significant levels: ** = *p*≤ 0.01; * = *p*≤ 0.05; and ns= non-significant. Error bars represent ± 1SD.

Wheat grown as mixtures accumulated greater amounts of N in the shoots compared to the sole-cropped wheat. For example, at maturity, wheat cv. ‘Alderon’ accumulated 41.5% more N in the shoots when grown as a mixture than as a sole crop together with weeds (Figure 2, Table 1). In contrast, the plant N pools of the legumes were higher in the sole crops than in the mixtures at both flowering and maturity (Table 1). At flowering, this translated to 55.1% and 6.4% greater N pool in sole crops of Faba bean cv. ‘Boxer’ and cv. ‘Fuego’, respectively, compared to growth in the mixed setup without weed competition. Based on the calculated NAE components (Figure 3), N uptake efficiency (U_N_) followed a similar trend as the accumulated N in the biomass. The high U_N_ of wheat in the mixture was associated with more biomass and N accumulation in the shoots than in the sole crop, while in the legumes, high U_N_ in the sole crop was associated with more biomass and N accumulation in the sole crop than in the mixtures. The grain specific N efficiency (E_N,g_), which represents the N conversion aspect of the plant, and grain N concentration (C_N,g_) were similar in the sole crops and mixtures of both wheat and faba bean.

The specific cultivar of the legume used influenced biomass and shoot N accumulation at flowering (Table 1), while the presence of the weed *C. album* reduced the N pool only for the wheat at maturity. There was no effect of weeds on any of the NAE components, as similar U_N_, E_N,g_ and C_N,g_ were found in all cases (Figure 3, Table S1). At both crop flowering and maturity, wheat accumulated more root N when grown in a mixture than as a sole crop (*p* < 0.000). In contrast, the legumes had higher root N in the sole crop than when grown as a mixture at both sampling periods (*p* < 0.000). The cultivar variable only had marginal effects on root N accumulation in the mixtures (*p* = 0.057 and *p* = 0.054 at flowering and maturity, respectively).

### 3.2. The Reduction in Plant N Pools in the Mixtures Is Correlated with Weed Biomass Accumulation

**Figure 4:**
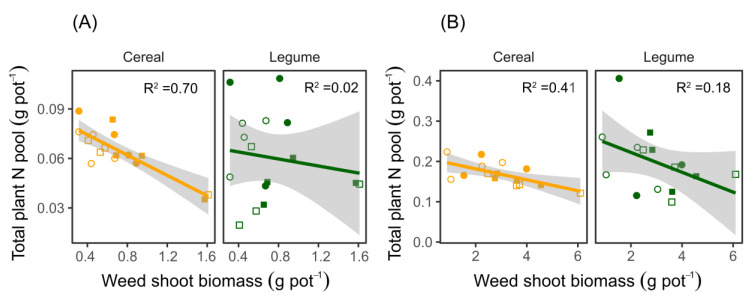
Relationships between total plant N pools and weed shoot biomasses for the cultivars of cereals and legumes pot-grown in mixtures and with the weed *C. album*. The plants were sampled at (**A**) flowering and (**B**) maturity. Both cultivars of cereals and legumes were considered in the analysis. Open and closed square symbols represent ‘Boxer’–‘Alderon’ and ‘Boxer’–‘Diskett’ cultivar mixtures, respectively, while open and closed circles represent ‘Fuego’–‘Alderon’ and ‘Fuego’–‘Diskett’ cultivar mixtures, respectively. Lines indicate linear regressions at crop flowering: cereals y= 0.087–0.031x and legumes y = 0.068–0.011x with *p* ≤ 0.001 and *p* = 0.567, respectively; and at maturity: cereals y = 0.21–0.014x and legumes y = 0.27–0.025x with *p* < 0.0098 and *p* = 0.108, respectively.

**Figure 5:**
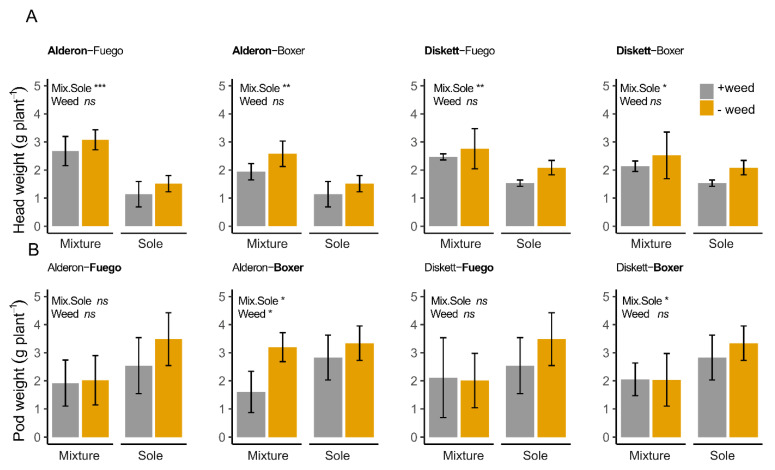
The mean (**A**) head height for wheat and (**B**) pod weight for two faba bean cultivars pot-grown as sole crops and in mixture, with and without the weed *C. album* at maturity. The error bars represent ±1 SD of the different cultivar combinations. The cultivars whose names appear in bold correspond to the ones presented in the graph. Symbols show ANOVA results with significance levels: *** = *p* ≤ 0.001; ** = *p* ≤ 0.01; * = *p* ≤ 0.05; and ns= non-significant.

The total plant N pools in the cereals grown in the mixtures decreased with increasing amounts of biomass accumulated by the weeds at both flowering and maturity. (Figure 4A,B; *p* = 0.001 and *p* < 0.009 for flowering and maturity, respectively.) The crop cultivars used in the mixtures did not influence the relationships between the plant N pools and accumulated shoot biomass in *C. album*. Similarly, growing weeds in the same pots with the legumes did not significantly reduce the plant N pools for the legumes at both flowering and maturity (flowering; R^2^ = 0.02, *p* = 0.567 and maturity; R^2^ = 0.18, *p*= 0.108). Despite the reduction in plant N pools of the cereals, the cereal yield, assessed as head weight, was not affected by weed growth as no difference was found between crops grown with and without the weed (*C. album*). All cereal cultivars grown in the mixtures had higher yields than the same cultivars grown as sole crops (Figure 5A, Table S2). The greatest yield difference was observed in the wheat cv. ‘Alderon’, where 50.7% greater head weight was observed when intercropped with faba bean cv. ‘Fuego’ in the absence of weed competition. For legumes, ‘Boxer’ in a ‘Boxer’–‘Alderon’ mixture grown without *C. album* produced a higher yield (pod weight) than when grown with *C. album* (Figure 5B, Table S2). The yield-related loss attributed to weed competition was 1.59 g plant^−1^, equivalent to a 49.8% lower yield under weed competition. Similarly, only ‘Boxer’ both in mixtures with ‘Alderon’ and ‘Diskett’ had yields higher in the mixture than the sole crop.

### 3.3. Plant Neighbor Affects the Accumulation of Other Nutrients (Ca, K, Mg and S) Relative to the Accumulation of N and P

We found a higher scaling exponent for nutrient concentrations in the legumes than the cereals, implying the relative accumulation of nutrients other than N and P (i.e., Ca, K, Mg, S) was greater in the legumes than the cereals (Table 2). Crop mixing significantly decreased the scaling exponent of the cereals compared to growing them as sole crops, implying less accumulation of other nutrients (Ca, K, Mg and S) in relation to the accumulation of N and P (*p* = 0.016, Table 3). Growing the weed *C. album* in the same pots significantly increased the scaling exponents of the legumes compared to growing the same legumes in the absence of the weed (*p* = 0.005). The scaling exponents did not significantly differ between the cultivars.

## 4. Discussion

The point of departure for this study was the set of results from a field experiment in which the same wheat and faba bean cultivars were grown as sole crops and in mixtures for two years [10], but with the difficulties associated with field studies, such as the limited possibilities to control growth conditions (e.g., plant neighbors, including weeds) and the consideration of below-ground plant parts. This investigation therefore complements the field study by Ajal et al. [10] and provides additional insights into the mechanisms underlying plant–plant interactions, especially with regard to nutrient accumulation patterns, including nutrients other than N. Furthermore, by linking plant functional traits to plant diversity and ecosystem processes, our research contributes to the further understanding of the mechanisms underpinning plant community productivity, stability and nutrient dynamics, and thus to biodiversity–ecosystem function (BEF) theory development, for which a clear mechanistic understanding is still lacking [24,25].

### 4.1. Effect of Diversity on N Accumulation Efficiency and Plant Biomass: Do Cultivars Matter?

We observed the contrasting effects of diversity on shoot N accumulation by wheat and faba bean, with the wheat and faba bean having higher shoot N pools in the mixtures and sole crops, respectively. Plant–plant interactions between crop species with different functional trait values facilitate complementarity, which ensures a more complete resource use for the components in the mixture [26]. Furthermore, the spatial distribution of plants or temporal availability of resources ensures that a more complete resource capture is achieved by the plants in the community [27]. In line with the above, our results were consistent with hypothesis *H1*. More biomass and N accumulation of the cereals in the mixture than sole crop were associated with a higher N uptake efficiency (U_N_), which suggests that the mixtures had accumulated more plant N than the sole crops. Although contrary to the cereals, the legume performance supports the results from several other studies that have shown the competitive disadvantage of legumes, especially when in interaction with cereals [6,10,28]. Compared to the legumes, cereals can achieve a more developed root system at a faster rate, especially in the early stages of crop development, which enables the exploration of larger soil volumes for the uptake of resources such as water and nutrients [16,29,30]. Ideally, a high N acquisition rate, as we observed in cereals that were grown in mixtures, would need to be complemented by a high conversion efficiency of the acquired N in order to maximize grain yield. However, the yield increase in the mixture was exclusively explained by N acquisition since we found no evidence for enhanced N conversion efficiency (represented by grain specific N efficiency, E_N,g_, in our study) in the mixtures. The high accumulation of shoot N pool in the legume sole crops may be explained by a low competitive pressure due to the absence of a highly competitive plant neighbor (wheat), and the reliance on N fixation to meet part of its N requirement. The faba bean cultivar used, either in the sole crop or mixture, influenced the amount of N pools accumulated both above- and below-ground at flowering and maturity. Although we do not have any empirical evidence for the cultivar differences, a plausible explanation could be related to the differences in biological N fixation, which ensured that a different amount of N was utilized by the crop during the growth period as was, for example, illustrated in the pea by Hauggaard-Nielsen et al. [31]. The differences between the cultivars of faba bean may have implications for management, especially in relation to cultivar selection, when designing a mixed crop system. Although we here focused on cultivar differences for plant N pools, the same trend may also apply for N uptake or leaf area index (LAI), because N is known to enhance plant leaf and canopy development [32]. Incidentally, canopy structure is one of the traits that can be manipulated to allow maximum light interception in crop mixtures [33]. Therefore, farming practitioners could consider more functionally different canopy characteristics when choosing components to include in mixtures because they, together with other factors, influence radiation use efficiency (RUE). As expected, we found that the shoot N pool increased between flowering and maturity in both cereals and legumes. However, in a few cases, the N pools of the cereals were similar or even decreased between flowering and maturity when the same cultivars were grown in the field [10]. From the onset of crop flowering to maturity, many physiological changes occur that relate to N remobilization from the leaves or N re-allocation to the grains, which may be influenced by various factors. For example, the time of leaf senescence (i.e., delayed senescence), which favors prolonged photosynthesis, and the high N stored at flowering can favor more grain N accumulation at maturity [34]. 

### 4.2. Weed Influences N Accumulation Efficiency in Wheat–Faba-Bean Mixtures

The diversity in crop mixtures, especially when accomplished by species or cultivars with great differences in functional traits, may facilitate more efficient resource use, which is associated with the complementary sharing of resources from a common pool [5,35]. In such cases, complementarity or facilitation supersedes interspecific competition between the components of the mixture. Competition from weeds is undesirable and associated with the reduction in the quality or quantity of yield [36,37]. In the case of this study, competition from the weed *C. album* resulted in less accumulated plant N pools in the crops at both flowering and maturity, which is consistent with our hypothesis *H3*. It is likely that most of the N that the wheat and faba bean were deprived of, or did not take up, constituted the portion accumulated by the weeds for biomass production. This is based on the assumption that the crop and weeds draw resources from the same resource pool given that resource pool similarity or diversity can have different influence on crop–weed competition intensity [38]. The lower plant N accumulation by the crop plants when grown together with *C. album* is consistent with the results by Jäck et al. [7], in which accumulated N by the weeds (*C. album*) was positively correlated and increased with increasing N available to the weeds, although the study was based on a pea–barley mixture. It should also be noted that *C. album* is a highly responsive weed in relation to N availability [7]. These two factors taken together may explain the observed relationship between the total plant N pools of the crop plants and the biomass accumulation of the weeds. However, in general, the competition from *C. album* was not strong enough to compromise the crop yield, except in the faba bean cv. ‘Boxer’. We think this was associated with the weak competition between the crop and the weed for above-ground resources. Under controlled conditions (e.g., pot experiments), where interaction is usually among few plants, individual species in the interaction may efficiently utilize light (hence light becomes non-limiting for growth), and stronger competition for resources, such as water and nutrients, is likely to occur below- than above-ground [39]. In the field, changes in the canopy structure make competition for light between the crop and weed an important element of the interaction, which is significant in the suppression of weeds. Therefore, the better performance of all cereal cultivars in the mixtures compared to the sole crops, irrespective of whether in interaction with the weed or not, is due to the higher competitiveness of the cereals than the legumes. Cereals generally have faster initial growth than the legumes under similar growth conditions and possess more developed and deeper root systems that favor greater resource uptake.

### 4.3. How Does N and P Uptake Relate to Other Major Nutrients? Does Mixing Improve the Accumulation of the Other Nutrients?

Compared to other nutrients, N and P have been extensively studied, which is attributed to their role as being the most limiting for plant growth [13]. The relative accumulation of nutrients other than N and P, especially in cereals and legumes grown as sole crops and mixtures, provided new insights for nutrient balance as a result of species interactions. Through this study, we showed that the specific cultivar or plant species used significantly affects the nutrient accumulation and stoichiometry of the neighboring plants grown together in the same pot. We expected the scaling exponents for wheat and faba bean to differ when grown in sole crop or mixture (hypothesis *H4*). Indeed, the cereals in the mixture (with legumes) significantly decreased their scaling exponent, implying that the interaction with the legumes reduced the relative accumulation of nutrients other than N and P. The cereals rely on N and P accumulation for important growth processes, such as tillering (N) [40] and root development and energy metabolism (P) [41]. Therefore, it is not surprising that, when the accumulation of other nutrients increased at a lower rate, more N and P were accumulated. Although we found no significant variation in the scaling exponents of faba bean grown in sole crop and mixture, the scaling exponent of faba bean (legume) was generally higher than that of wheat (cereal). The difference in the crop species scaling exponents implies that the relative accumulation of nutrients other than N and P was greater in the legumes than the cereals. We attribute this to the N-fixing nature of the legumes, which promotes the reliance on fixed N to meet part of its own N requirement. Thus, the increase in demand (and accumulation) of other nutrients is more important for the legumes than the cereals [17]. In the presence of weed competition, weeds pose a strong competition for N with the legumes, e.g., as earlier illustrated for pea in competition with *C. album* [7]. Since the legumes often and to a high degree rely on N from atmospheric N fixation, the need to accumulate other nutrients might become more important than accumulating N. In addition, it has been shown in another study that weed mixtures (including *C. album*) can facilitate the greater uptake of P due to the mycorrhizal colonization of the roots [19]. This may partly explain why in our study the scaling exponents of the legumes increased in the presence of weeds. It is worth highlighting the magnitude of scaling exponents (scaling exponent >2) of the legumes in the mixture or when grown together in a mixture with the cereals and weed, which resulted in a strong and significant effect of the neighboring plant on nutrient stoichiometry. This observation is particularly important as it may have implications for nutrient (co-) limitation as shown earlier by Weih et al. [15]. The co-limitation may be applicable in this study since having a legume or a weed as a neighboring plant may result in more accumulation of one or more nutrients other than N and P, but may also be applicable in the field, especially when crop mixtures are grown. However, the observed effects of plant neighbor on nutrient stoichiometry must be validated in field experiments.

### 4.4. Implications for Field Conditions and Management

This study was based on species combinations that have previously, under a different set of objectives, been tested under field conditions [7,10]. We conducted this study under semi-controlled conditions, specifically to enable us to sample both above- and below-ground plant parts. Interestingly, some trends were consistent with the findings of the field studies by Ajal et al. [10] and Jäck et al. [7], for example, the relationship between crop and weed N accumulation. However, as in the case of many controlled studies, concerns of their applicability under field conditions often arise. Nevertheless, the findings from this study are important in making informed decisions when designing crop mixtures. Specifically, the difference in N accumulation and biomass in the shoots of different faba bean cultivars suggests the need to focus on specific cultivars with traits that enhance nutrient accumulation efficiency. The increased N pools between flowering and maturity may also have implications for nutrient management. This suggests that split application should be considered, with one application performed relatively late in the season (for example, around booting of the cereals) to facilitate more N accumulation at a later stage during the growth period, and translocation to the grains for higher quality products.

### 4.5. Study Limitations

The number of cereal and legume cultivars in this study was limited to only two cultivars per species, which limited the potential to explore the scaling exponent variability compared to if more cultivars had been used. We also estimated the dry weight and plant N pools of the roots based on the assumption that their proportions above- and below-ground are similar when grown as sole crop and in mixture. We acknowledge that, although this enabled us to estimate root characteristics, the plasticity of traits may be influenced by the different growth conditions in the mixtures compared to the sole crops. The mentioned limitations notwithstanding, we think this study generated valuable information for nutrient accumulation and species interaction in crop mixtures.

## 5. Conclusions

This study demonstrated some mechanistic links explaining why species and cultivar choice is important for resource limitation, plant-internal resource allocation and use and, ultimately, the growth performance of plants grown in mixtures with and without the presence of co-existing weeds. Crop interaction with weeds (*C. album*) results in reduced amounts of N pools accumulated by the crop plants, especially wheat, a mechanism similar to the effect of cereals on legumes when the two are grown together. These interactions are strongly modulated by the characteristics of the crop cultivars used in the mixture. Results from the analysis of stoichiometric scaling exponents show that the relative accumulation of other nutrients (Ca, K, Mg and S) by the crops compared to the accumulation of N and P depends on which plant neighbors the focal plant has. Since this study involved different crop species, cultivars and crop interaction with weeds, it provides new insights necessary for evidence-based species selection when designing crop mixtures. Future plant physiological studies on the mechanisms underlying the differential accumulation of nutrients when grown with different neighbors are important for our understanding of plant–plant interactions. Furthermore, studies involving specialized techniques for quantifying below-ground interaction in mixtures, for example, DNA-based methods or Infrared Spectroscopy, could provide more precise measures for quantifying root interactions between different species in the mixture than was possible with the method used in our study.

## Figures and Tables

**Table 1:** Analysis of variance for plant nitrogen pools in the shoot and roots of different cultivars of wheat and faba bean grown as sole crops and mixtures sampled at flowering and maturity. Symbols show results with significant levels: *** = *p* ≤ 0.001; ** = *p* ≤ 0.01; * = *p* ≤ 0.05.

Factor	Flowering		Maturity	
	Cereal	Legume	Cereal	Legume
Shoot				
Weed	0.2028	0.4512	0.0246 *	0.1430
Cultivar	0.4714	<0.0001 ***	0.5768	0.7630
Mix.Sole	<0.0001 ***	0.0006 ***	<0.0001 ***	0.0008 ***
Weed×Cultivar	0.4912	0.4661	0.9034	0.3167
Weed×Mix.Sole	0.5915	0.2649	0.4683	0.1726
Cultivar×Mix.Sole	0.4418	0.3256	0.0893	0.7012
Weed×Cultivar×Mix.Sole	0.8027	0.4774	0.5317	0.2482
Root				
Weed	0.0080 **	0.0190 *	0.1781	0.0645
Cultivar	0.9514	<0.0001 ***	0.0870	0.0017 **
Mix.Sole	<0.0001 ***	<0.0001 ***	<0.0001 ***	<0.0001 ***
Weed×Cultivar	0.3825	0.9960	0.5227	0.1756
Weed×Mix.Sole	0.0012 **	0.1450	0.3454	0.0513
Cultivar×Mix.Sole	0.8327	0.8606	0.2458	0.0297*
Weed×Cultivar×Mix.Sole	0.6327	0.8662	0.6260	0.5824

**Table 2:** Scaling exponents (α) for cereals and legumes grown as sole crops and in mixtures, with and without the weed *C. album*, based on the nutrient volumes for N and P (VNP) vs. the volumes of other nutrients (VOth; K, Ca, Mg and S). The scaling exponents were based on data from the flowering stage.

Group	Scaling Exponent (α)	N	SE
Cereals sole	1.349	8	0.026
Legumes sole	2.001	8	0.012
Cereals mix	1.284	16	0.012
Legumes mix	2.033	16	0.025
Cereals + Weed	1.329	8	0.034
Legumes + Weed	2.077	8	0.019

**Table 3:** Pairwise comparison of scaling exponents following analysis of variance for cereals and legumes grown as sole crops and mixtures, with and without the *C. album* weed. The scaling exponents were based on data from the flowering stage. Symbols show results with significant levels: *** = *p* ≤ 0.001; ** = *p* ≤ 0.01; * = *p* ≤ 0.05.

Comparison	F-Value	*p*-Value
Cereals sole vs. Legumes sole	518.42	0.000 ***
Cereals sole vs. Cereals mix	6.85	0.016 *
Cereals sole vs. Cereals + Weed	0.22	0.648
Legumes sole vs. Legumes mix	0.76	0.393
Legumes sole vs. Legumes + Weed	11.44	0.005 **
Cereals mix vs. Legumes mix	729.52	0.000 ***
Cereals + Weed vs. Legumes + Weed	368.82	0.000 ***

## Data Availability

All data supporting results in this paper are openly accessible at http://doi.org/10.6084/m9.figshare.19607040.

## Supplementary Materials

The following supporting information can be downloaded at: https://www.mdpi.com/article/10.3390/biology11050630/s1, Figure S1: Daily precipitation, average temperature and relative humidity for Ultuna near Uppsala, Central Sweden, during the period of experimentation in 2019. The arrows show the time points for planting, and destructive samplings at flowering and maturity. Relative humidity and air temperature data were recorded using a weather logger installed in net yard. Rainfall data were accessed from Ultuna Climate Station, Uppsala (Sweden); Table S1: Analysis of variance for Nitrogen Accumulation Efficiency (NAE) components and shoot dry weight at flowering for different cultivars of cereals and legumes. The different cultivars (Cultivars) were grown as sole crops and in mixtures (Mix.Sole), with and without *C. album* (Weed); Table S2: Analysis of variance for yield assessed as head weight and pod weight in the cereals and legumes, respectively. Analysis was performed for different cultivar combinations pot-grown in sole crops and mixtures (Mix.Sole), with and without the weed C. album (Weed).
