# Supplementary material for: Nutrient Accumulation Pattern in Mixtures of Wheat and Faba Bean Is Strongly Influenced by Cultivar Choice and Co-Existing Weeds
Source: Biology (Basel). 2022 Apr 20;11(5):630. doi: 10.3390/biology11050630 (PMC9137686; doi:10.3390/biology11050630)
Supplement: Supplementary file 1 [file biology-11-00630-s001.zip › biology-1653686-supplementary.pdf]

biology

MDPI

Article

Nutrient Accumulation Pattern in Mixtures of Wheat and Faba Bean Is Strongly Influenced by Cultivar Choice and Co-Existing Weeds

James Ajal * and Martin Weih

Department of Crop Production Ecology, Swedish University of Agricultural Sciences, P.O. Box 7043, SE-75007 Uppsala, Sweden; martin.weih@slu.se
* Correspondence: james.ajal@slu.se; Tel.: +46702157239

Supplementary Information

Table S1. Analysis of variance for Nitrogen Accumulation Efficiency (NAE) components and shoot dry weight at flowering for different cultivars of cereals and legumes. The different cultivars (Cultivars) were grown as sole crops and in mixtures (Mix.Sole), with and without *C. album* (Weed). Symbols show ANOVA results with significance levels;*** = P≤ 0.001; ** = P≤ 0.01; * = P≤ 0.05.

.	Cereals		Legumes	
	F-value	p-value	F-value	p-value
**N uptake efficiency (U_N)**				
Weed	6.26	0.0666	3.95	0.1179
Cultivar	3.92	0.1187	6.79	0.0597
Mix.Sole	33.35	0.0045**	18.27	0.0129*
Weed × Cultivar	0.17	0.7053	1.01	0.3723
Weed × Mix.Sole	0.31	0.6080	2.19	0.2134
Cultivar × Mix.Sole	0.01	0.9451	0.05	0.8399
Weed × Cultivar × Mix.Sole	0.12	0.7457	1.28	0.3211
**Grain specific N efficiency ($E_{N,g}$)**				
Weed	2.12	0.2192	1.29	0.3195
Cultivar	0.44	0.5427	3.59	0.1312
Mix.Sole	4.11	0.1126	1.90	0.2397
Weed × Cultivar	0.59	0.4852	0.27	0.6280
Weed × Mix.Sole	0.86	0.4055	0.15	0.7175
Cultivar × Mix.Sole	3.88	0.1203	0.15	0.7210
Weed × Cultivar × Mix.Sole	0.08	0.7866	0.80	0.4205
**Grain N concentration ($C_{N,g}$)**				
Weed	0.60	0.4818	0.86	0.4071
Cultivar	0.60	0.4818	2.72	0.1743
Mix.Sole	1.93	0.2376	1.23	0.3293
Weed × Cultivar	1.41	0.3007	0.36	0.5787
Weed × Mix.Sole	3.07	0.1545	3.42	0.1381
Cultivar × Mix.Sole	4.80	0.0936	0.00	0.9787
Weed × Cultivar × Mix.Sole	0.64	0.4668	0.01	0.9361
**Shoot dry weight**				
Weed	1.17	0.3406	0.57	0.4906
Cultivar	3.61	0.1302	14.01	0.0201*
Mix.Sole	19.20	0.0119*	13.75	0.0207*

Weed × Cultivar	1.01	0.3726	0.87	0.4030
Weed × Mix.Sole	0.43	0.5491	0.49	0.5224
Cultivar × Mix.Sole	1.19	0.3363	0.12	0.7460
Weed × Cultivar × Mix.Sole	0.07	0.8083	0.38	0.5708

Table S2. Analysis of variance for yield assessed as head weight and pod weight in the cereals and legumes, respectively. Analysis was done for different cultivar combinations pot-grown in sole crops and mixtures (Mix.Sole), with and without the weed *C. album* (Weed). Symbols show results with significance levels;*** = P≤ 0.001; ** = P≤ 0.01; * = P≤ 0.05.

Cultivar combinations	Cereals		Legumes	
	F-value	p-value	F-value	p-value
**Alderon-Fuego**				
Mix.Sole	58.11	0.0003***	5.23	0.0622
Weed	3.66	0.1516	1.31	0.3354
Mix.Sole × Weed	0.00	0.9472	0.85	0.3916
**Alderon-Boxer**				
Mix.Sole	26.61	0.0021**	7.23	0.0361*
Weed	7.88	0.0674	17.26	0.0254*
Mix.Sole × Weed	0.54	0.4911	4.58	0.0762
**Diskett-Fuego**				
Mix.Sole	24.96	0.0025**	2.99	0.1343
Weed	7.01	0.0772	0.58	0.5004
Mix.Sole × Weed	2.17	0.1908	0.90	0.3792
**Diskett-Boxer**				
Mix.Sole	6.16	0.0476*	8.04	0.0297*
Weed	2.78	0.1942	0.45	0.5497
Mix.Sole × Weed	1.01	0.3539	0.52	0.4974

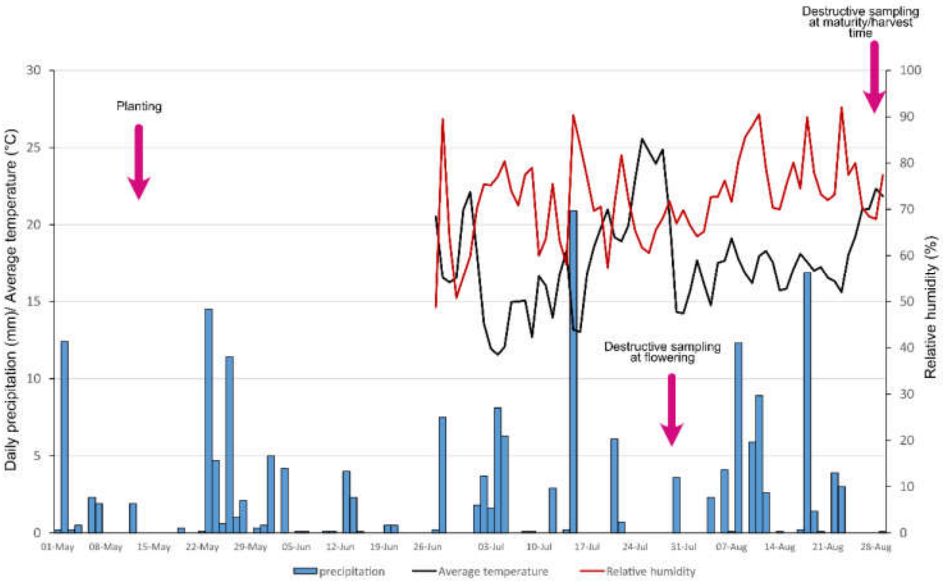

Figure S1. Daily precipitation, average temperature and relative humidity for Ultuna near Uppsala, Central Sweden, during the period of experimentation in 2019. The arrows show the time points for planting, and destructive samplings at flowering and maturity. Relative humidity and air temperature data were recorded using a weather logger installed in net yard. Rainfall data was accessed from Ultuna Climate Station, Uppsala (Sweden).
